# Culturing the Plastisphere: comparing methods to isolate culturable bacteria colonising microplastics

**DOI:** 10.3389/fmicb.2023.1259287

**Published:** 2023-10-03

**Authors:** Emily M. Stevenson, Angus Buckling, Matthew Cole, Penelope K. Lindeque, Aimee K. Murray

**Affiliations:** ^1^Faculty of Health and Life Sciences, European Centre for Environment and Human Health, Environment and Sustainability Institute, University of Exeter Medical School, Penryn Campus, Cornwall, United Kingdom; ^2^Marine Ecology and Biodiversity, Plymouth Marine Laboratory, Plymouth, United Kingdom; ^3^College of Life and Environmental Sciences, University of Exeter, Penryn Campus, Cornwall, United Kingdom

**Keywords:** biofilm, microplastics, Plastisphere, bacterial culture, biofilm extraction, microbiology

## Abstract

Microplastics quickly become colonised by diverse microbial communities, known as the Plastisphere. There is growing concern that microplastics may support the enrichment and spread of pathogenic or antimicrobial resistant microorganisms, although research to support the unique role of microplastics in comparison to control particles remains inconclusive. Limitations to this research include the microbiological methods available for isolating adhered microbes. Culture-based methods provide some of the most established, accessible and cost-effective microbiological protocols, which could be extremely useful in helping to address some of the remaining key questions in Plastisphere research. Previous works have successfully cultured bacteria from plastics, but these have not yet been reviewed, nor compared in efficiency. In this study, we compared four common biofilm extraction methods (swabbing, sonication, vortexing, sonication followed by vortexing) to extract and culture a mixed community of bacteria from both microplastic (polyethylene, polypropylene and polystyrene) and control (wood and glass) particles. Biofilm extraction efficiency and viability of bacterial suspension was determined by comparing CFU/mL of four different groups of bacteria. This was verified against optical density and 16S rRNA qPCR. Overall, we found that all tested methods were able to remove biofilms, but to varying efficiencies. Sonicating particles with glass beads for 15 min, followed by vortexing for a further minute, generated the highest yield and therefore greatest removal efficiency of culturable, biofilm-forming bacteria.

## 1. Introduction

Microplastics (0.1 μm–5 mm; Thompson et al., 2004) are environmentally prevalent pollutants that can support a diverse array of microbes, known as the Plastisphere (Zettler et al., 2013). These microplastic-associated biofilms have also been shown to harbour pathogenic or antimicrobial resistant bacteria, potentially increasing human or animal exposure to disease-causing, drug-resistant microbes (Arias-Andres et al., 2018; Bowley et al., 2021). Therefore, it is crucial for research efforts to determine the risk posed by microplastics to ecological systems and human, animal or environmental health. However, it is not yet clear whether the Plastisphere includes significantly more bacteria of concern than naturally existing particles.

There are a number of different techniques that have been used to investigate the microbial community profiles associated with microplastics (De Tender et al., 2017; Bartkova et al., 2021). These methods can be divided into molecular (e.g., DNA extraction followed by downstream applications such as sequencing), and culture-based (e.g., cultivation on agar plates) techniques. Molecular methods can be used to provide a holistic and sensitive picture of community composition, virulence and/or antimicrobial resistance (AMR). The first step of almost all molecular protocols is the extraction of DNA from the community, and the efficiency of DNA extraction methods from microplastics were previously evaluated by Debeljak et al. (2017). Culture-based methods have also been adopted in previous Plastisphere research (Table 1), though, these methods have not yet been compared in efficiency.

**Table 1:** Summary of published studies which cultured biofilm extractions from plastics.

Biofilm extraction method	Culturing method	Plastic(s)	References
Enrichment	Luciferase expression	HDPE	Ormsby et al. (2023)
	Agar plates	Mixed fragments and pellets	Hernández-Sánchez et al. (2023)
		PA, PC, LDPE, PET, PP, PS, PVC	Tan et al. (2022)
		Sewage-associated plastic waste	Metcalf et al. (2022)
		LDPE, HDPE, PP	Munir et al. (2022)
		LDPE	Song et al. (2020)
		‘Nurdles’	Rodrigues et al. (2019)
		Mixed particles	Kirstein et al. (2016)
Homogenisation	Agar plates	Mixed microplastics	Zhang et al. (2020)
		Mixed plastics	Silva et al. (2019)
Scraping	Agar plates	PET, PS, PE, PU, PA, PP, PVC	Pang et al. (2023)
		PE	Syranidou et al. (2017)
		HDPE, LDPE, PP	Sudhakar et al. (2007)
		ABS	Junker and Hay (2004)
Shaking	Agar plates	PP	Liu et al. (2023)
Shaking and vortexing	Agar plates	Mixed plastics	Quilliam et al. (2014)
Shaking with glass beads	Agar plates	PET	Vidal-Verdú et al. (2022)
		PP	Szabó et al. (2021)
		PE	Lobelle and Cunliffe (2011)
Sonication	Agar plates	Mixed plastics	Liang et al. (2023)
		PET	Grogan et al. (2021)
Sonication & vortexing	Agar plates	PS	Perveen et al. (2023)
		Nylon and PS	Forero-López et al. (2022)
Swabbing	Agar plates	PET and PE	Kapetanović et al. (2023)
		Mixed plastics	Moore et al. (2020)
		HDPE	Joseph et al. (2001)
Vortexing	Agar plates	PE	Metcalf et al. (2023)
		Mixed plastics	Rasool et al. (2021)
		PS, PP, PLA	Morfin (2021)
		PS	Laganà et al. (2019)
Vortexing and scraping	Agar plates	PE	Zhurina et al. (2023)
Vortexing and sonication	Conjugation assay	PS	Arias-Andres et al. (2018)
	Agar plates	Acrylic	Kim and Han (2014)
		PMMA	Kobayashi et al. (2009)
Vortexing with glass beads	Agar plates	LDPE, HDPE, PP, PVC, PET	Lear et al. (2022)
		PE, PEVA, PP	Radisic et al. (2020)
		PE-aluminium-PE	Lehtola et al. (2004)
Washed with sterile seawater	Agar plates	PS	Carpenter et al. (1972)

ABS, acrylonitrile butadiene styrene; HDPE, high-density polyethylene; LDPE, low-density polyethylene; PA, polyamide; PC, polycarbonate; PET, polyethylene terephthalate; PEVA, poly(ethylene-vinyl acetate); PLA, polylactic acid; PMMA, poly(methyl methacrylate); PP, polypropylene; PS, polystyrene; PU, polyurethane; PVC, polyvinyl chloride.

Culture-based methods, though less sensitive than molecular methods and limited to the study of culturable organisms, are cost-effective, replicable and widely accessible (e.g., to less specialised, smaller or lower-income laboratories). Perhaps more importantly, working with cultured isolates allows measurement of key phenotypes, such as AMR and pathogenicity, that can otherwise only be inferred from sequence-based methods.

When investigating the Plastisphere, the first step in most culture-based methods is to remove the biofilm from the particle, creating a bacterial suspension. Whilst the methods that have previously been adopted to achieve this have shown success in biofilm removal (Table 1), there has been no measure of removal efficiency. In some cases, biofilms are not removed but are instead used to produce an enrichment culture, whereby particles harbouring biofilms are added to a sterile, nutrient rich broth. The resulting liquid culture is then used for downstream approaches. However, this method may arguably only select for strains capable of free-living growth, biasing the community against strictly biofilm-forming phenotypes. In addition, inducing a free-living phenotype may alter the expression of virulence factors or AMR that is associated with biofilm formation. Therefore, we have not included this style of isolation in our study, and have instead focused on entire biofilm extraction, with the aim of retaining viable cells.

Without assessment of removal efficiency, culture-based research efforts are not currently optimised, comparable or consistent. This study therefore aimed to compare the efficiency of some of the most used protocols to extract viable bacterial cells from microplastics. The methods selected for testing were: swabbing, sonication with glass beads, vortexing with glass beads, and sonication with glass beads followed by vortexing. In additional to testing removal efficiency from microplastics, natural (wood) and inert (glass) particles were also included, to ensure the extraction method we propose is the most efficient for both microplastic and control particles. This is crucial, given that future research should not only focus on the microbial communities present on microplastics, but also in comparison to communities present on natural control substrates.

## 2. Materials and equipment

### 2.1. Materials

Microplastic and wood particles sampled from the environment, 4 mm glass beads (Novagen ColiRollers, LOT: D00136263), Iso-Sensitest broth (Oxoid; LOT: 3177183), glycerol (ThermoFisher, LOT: P01H051), NaCl (Sigma-Aldrich, PCode: 1003326144), Luria-Bertani (LB) agar (Fisher Bioreagents, LOT: 188453), CM1205 Chromogenic Coliform agar (ISO; Oxoid, LOT: 3004362), DNeasy Ultra-Clean Microbial kit (Qiagen, LOT: 169011985), DNeasy PowerBiofilm kit (Qiagen, LOT: 169048148), custom synthetic gBlocks and primers (Table 2) provided by IDTDNA, PrimerDesign Precision Plus SYBR Green Master Mix (Z-PPLUS-R-SY-10ML) and bovine serum albumin (20 mg/mL, Fisher Scientific, LOT, 170419-0461).

**Table 2:** Primers and gBlocks used in this study for targeting 16S rRNA.

Forward primer (5′–3′)	Reverse primer (5′–3′)	Product size (bp)	gBlock sequence and length (bp)	References
CGGTGAATACGTTCYCGG	GGWTACCTTGTTACGACT	142	ACGGTGAATACGTTCCCGGGCCTTGTACACACCGCCCGTCACACCATGGGAGTGGGTTGCAAAAGAAGTAGGTAGCTTAACCTTCGGGAGGGCGCTTACCACTTTGTGATTCATGACTGGGGTGAAGTCGTAACAAGGTAACCG – (144)	gBlock: (Murray et al., 2018, 2020)
				Primers: (Suzuki et al., 2000)

### 2.2. Equipment

PerkinElmer 208 Spotlight 400 (Perkin Elmer, United Kingdom), CAT-II safety cabinet, sterile cotton swab (DeltaLab, LOT: 191114), vortex (Scientific Industries, serial number A6. 1130), sonication bath (VWR Ultrasonic Cleaner, Model: USC100T), Varioskan Flash plate reader (serial number 3001-1778), BioTek Synergy plate reader (serial number 254.462), colony counter (Stuart, serial number RCC0221P160) and Applied Biosystems QuantStudio 7 Flex (serial number 278871498).

## 3. Methods

### 3.1. Particle preparation

#### 3.1.1. Sampling particles

Microplastic and wood particles were collected by-hand in October 2021 from a riverbank in Truro, Cornwall (United Kingdom; 50.260048, −5.045549). Microplastics were separated into sterile 50 mL falcon tubes using forceps, with a separate tube for ‘bio-beads’ (bio-media from sewage treatment), ‘nurdles’ (pre-production pellets), expanded polystyrene beads and wood. All samples were transported in cool boxes and processed within 4 h. In the laboratory, all particles were rinsed in deionised water to remove any remaining sediment or natural debris from the surface, then left to air dry. Microplastic and wood particles that were 4 mm in size were selected for further processing. Additionally, 4 mm glass beads (Novagen ColiRollers, LOT: D00136263) were purchased.

#### 3.1.2. Particle sterilisation

Bio-beads, nurdles, glass beads and wood particles were autoclaved at 121°C for 15 min. To validate sterility, 6 of each particle were placed into sterile 15 mL falcon tubes containing 10 mL Iso-Sensitest broth (Oxoid; LOT: 3177183) and shaken (180 rpm) at 37°C overnight. Sterility was confirmed for all particles where there were no visual changes in optical density (OD) of the media. As the polystyrene particles were expanded polystyrene, autoclaving was not suitable for sterilisation. Therefore, gamma irradiation was outsourced to Becton Dickinson (Plymouth, United Kingdom): 500 polystyrene particles were separated into 5 mL Eppendorf tubes and gamma irradiated (10.2–10.6 kGy delivered for 3,600 s x2, followed by a further 900 s). As before, to validate sterility, 6 of the particles were placed into 10 mL Iso-Sensitest broth and shaken (180 rpm) at 37°C overnight. Sterility was confirmed for all particles where there were no visual changes in optical density (OD) of the media.

#### 3.1.3. Attenuated total reflectance Fourier-transform infrared spectroscopy

To ensure all nurdles, bio-beads and polystyrene particles were the same polymer type within their morphotypes, we performed attenuated total reflectance Fourier-transform infrared spectroscopy (ATR-FTIR) using a PerkinElmer 208 Spotlight 400 (Perkin Elmer, United Kingdom). Spectra were recorded as a mean of 4 scans at 4,000 nm wavelengths. Samples which produced spectra with a match less than 60% were automatically excluded. From this, bio-beads were identified as polyethylene and nurdles were identified as polypropylene. Any bio-beads or nurdles that did not match these polymers were excluded. As the machine applied force when scanning the particle, the shape of the polystyrene particles changed following identification. We therefore analysed a subset of these particles to ascertain that the remaining particles are highly likely to be expanded polystyrene.

### 3.2. Comparing biofilm extraction protocols

#### 3.2.1. Study particles

Particles (bio-beads/polyethylene, nurdles/polypropylene, expanded polystyrene, wood and glass) were sampled and processed as described (see section 3.1).

#### 3.2.2. Particle inoculation

**Figure 1:**
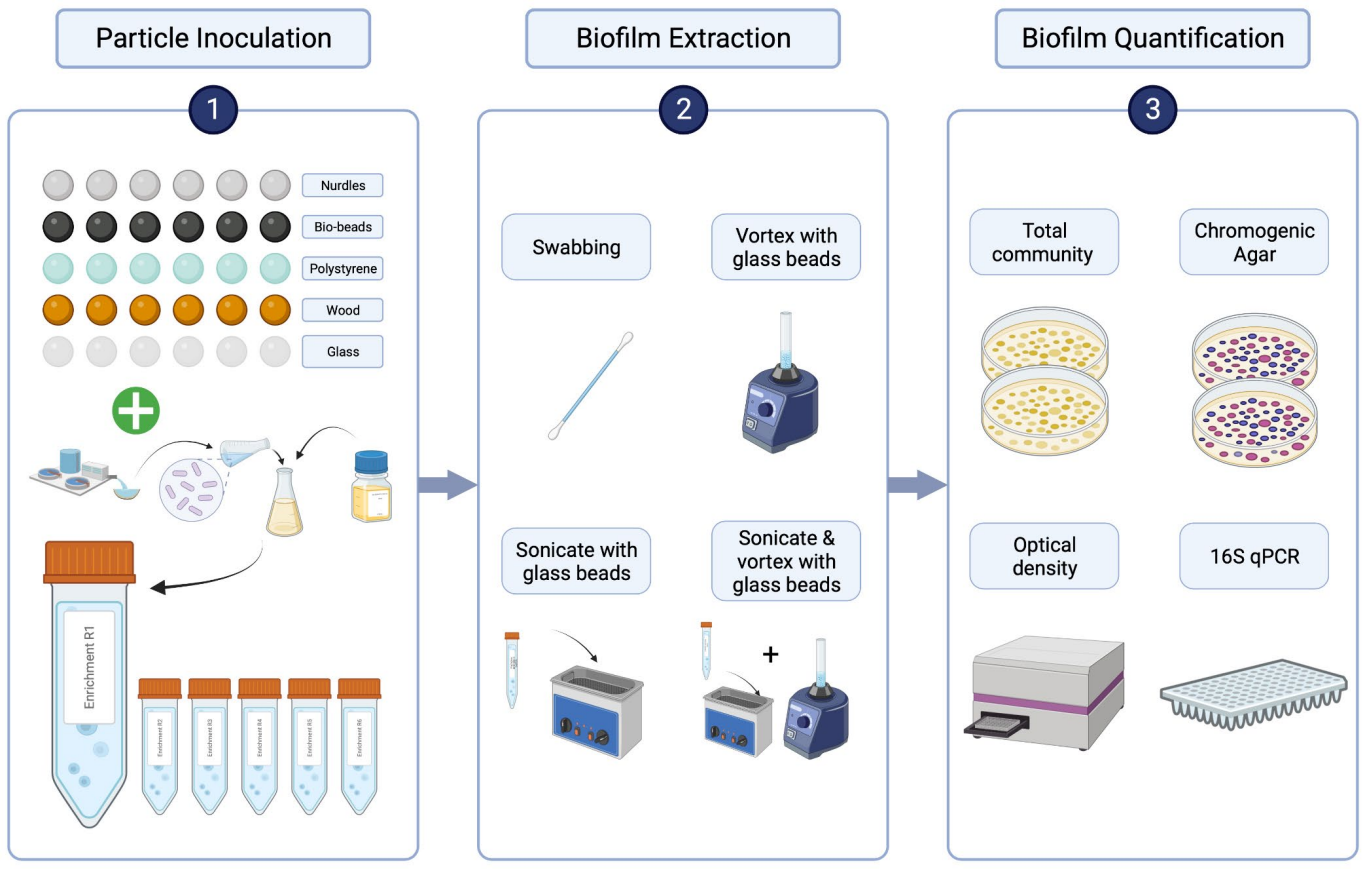
Comparing biofilm extraction protocols workflow. 1. Particles were inoculated with a washed sewage community in a nutrient rich broth overnight [x5 for each extraction method (4) and molecular validation (1)]. 2. Particles were rinsed with sterile NaCl then each of the 4 extraction methods were used to suspend biofilms from each particle type in sterile NaCl. 3. Biofilm extractions were quantified by plating biofilm suspensions on LB and chromogenic selective agar. Colony counts were validated using OD and genotypic quantification: DNA was extracted directly from the 5th set of inoculated particles using the DNeasy PowerBiofilm Kit (Qiagen), and the DNA was extracted from the biofilm suspensions for each extraction method using DNeasy UltraClean Microbial Kit (Qiagen). From these DNA samples, 16S rRNA quantitative-polymerase chain reaction (qPCR) was performed to assess the extraction efficiency of the biofilm extraction techniques compared with total biofilm community as obtained by the direct DNA extraction from the particles. Created with BioRender.com.

Sewage influent samples were collected from a wastewater treatment plant in Falmouth, United Kingdom (serving a population of approximately 43,000) in June 2021. Samples were transported in cool boxes, then mixed 1:1 with 40% glycerol (ThermoFisher, LOT: P01H051) and stored at −70°C until use. Aliquots were then thawed, spun down at 14,800 rpm for 1 min, supernatant discarded and pellet resuspended in 1 mL sterile 0.85% NaCl (Sigma-Aldrich, PCode: 1003326144) twice to remove chemical and nutrient carryover (Murray et al., 2020). The resuspended pellet was used to inoculate at 10% (vol/vol) in 10 mL Iso-Sensitest broth in 50 mL sterile falcon tubes containing sterile particles (Figure 1). Tubes were shaken at 50 rpm for 20 h at 37°C. Within each tube (i.e., biological replicate, of which there were 6), there were 6 individual particle replicates of each particle type, to account for individual variability of particle shapes and sizes (Figure 1). This inoculation procedure was repeated 5 times using the same starting inoculum to provide a set of biofilms for each of the extraction methods and a set for 16S rRNA qPCR.

#### 3.2.3. Biofilm extraction

**Figure 2:**
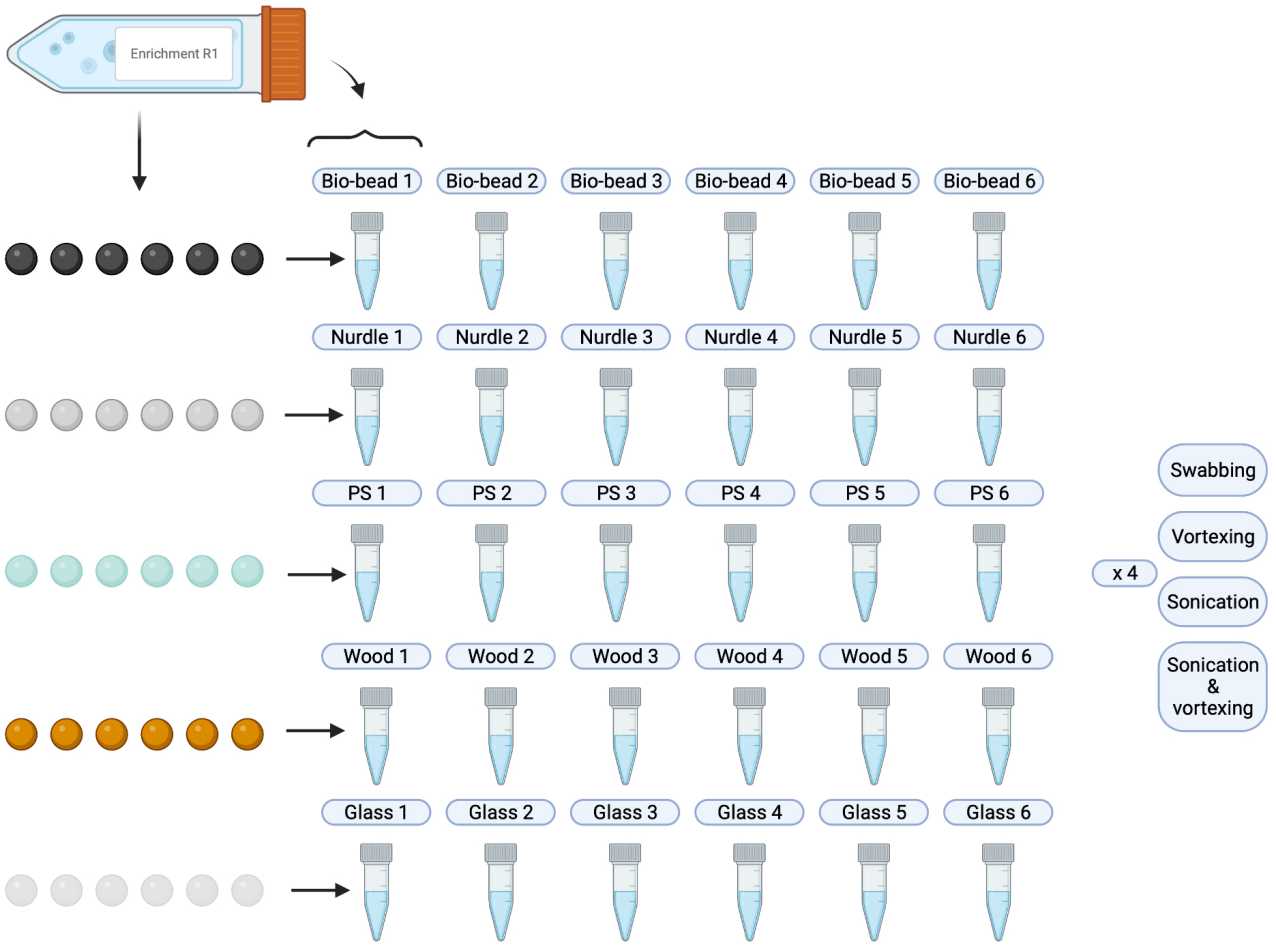
Schematic overview of biofilm suspensions produced. PS, polystyrene. Created with BioRender.com.

Following particle inoculation (see section 3.2.2), 500 μL of liquid culture was taken and cryogenically stored with glycerol. The remaining, surrounding liquid culture was decanted and particles were rinsed twice with sterile 0.85% NaCl to remove any loosely attached bacteria and left to air dry in sterile conditions inside a CAT-II safety cabinet. Each set of particles were then processed according to the different extraction methods, and biofilm suspension were produced as follows (Figure 2):

*Swabbing:* for each biological replicate and each particle type, individual particle replicates were secured using sterile forceps and, using a sterile cotton swab (DeltaLab, LOT: 191114), the entire surface of the particle was swabbed for 1 min. The cotton swab was then placed into an Eppendorf tube containing 600 μL sterile 0.85% NaCl and mixed in a circular motion for 10 s to encourage detachment of bacteria from the swab into the liquid.

*Vortexing with glass beads:* all 6 individual particle replicates for each of the 6 biological replicates for each particle type were placed into 600 μL sterile 0.85% NaCl in Eppendorf tubes. 5 sterile 4 mm glass beads were added to each tube and vortexed (2,500 rpm, Scientific Industries, serial number A6. 1130) for 1 min [as Lear et al. (2022)].

*Sonicating with glass beads:* all 6 individual particle replicates for each of the 6 biological replicates for each particle type were placed into 600 μL sterile 0.85% NaCl in Eppendorf tubes. 5 sterile 4 mm glass beads were added to each tube and placed into a sonication bath (VWR Ultrasonic Cleaner, Model: USC100T) for 15 min at 45 kHz.

*Sonication with glass beads, followed by vortexing:* all 6 individual particle replicates for each of the 6 biological replicates for each particle type were placed into 600 μL sterile 0.85% NaCl in Eppendorf tubes. 5 sterile 4 mm glass beads were added to each tube and placed into a sonication bath for 15 min at 45 kHz. Each tube was then vortexed (2,500 rpm, Scientific Industries, serial number A6. 1130) for 1 min.

From the suspensions from all the above methods, 100 μL was taken and used to generate OD (600 nm) readings, then diluted and plated onto agar plates. The remaining 500 μL of biofilm extraction was taken from the tube and placed into a fresh Eppendorf containing 500 μL 40% glycerol and stored at-70°C until DNA extraction.

*DNA extraction directly from the biofilm*: all 6 individual particle replicates for each of the 6 biological replicates for each particle type were placed into 1 mL 20% glycerol and stored at −70°C until DNA extraction.

#### 3.2.4. Biofilm quantification

##### 3.2.4.1. Optical density

OD readings were taken at 600 nm using either a Varioskan Flash (serial number 3001-1778) or BioTek Synergy (serial number 254.462). Absorbance values were not significantly affected by the model used (*p* > 0.05; Supplementary material 1.2), and only single timepoint measurements were obtained, thus reducing variability encountered by additional parameters, e.g., shaking speed and/or motion, and temperature variations. Sterile 0.85% NaCl was used for blanks/controls (6 replicates).

##### 3.2.4.2. Agar plates

To enumerate total culturable bacterial community, LB agar (Fisher Bioreagents, LOT: 188453) was used. To distinguish and enumerate *Escherichia coli* (*E. coli*), coliforms and non-coliforms, CM1205 Chromogenic Coliform agar (ISO; Oxoid, LOT: 3004362) was used. Biofilm suspensions were diluted prior to plating according to a 10-fold dilution series performed *a priori* to detect the optimum dilution which achieved 20–80 CFUs of *E. coli*. 100 μL of each diluted biofilm suspension was plated in duplicate onto both types of agar using glass beads (Worthington et al., 2001). Plates were inverted and incubated at 37°C for 18–24 h. Colonies were then counted using a colony counter (Stuart, serial number RCC0221P160) and CFU/mL was generated for each colony phenotype: total (LB), *E. coli* (blue/purple colonies), non-*E. coli* coliforms (pink colonies) and non-coliforms (white colonies).

##### 3.2.4.3. DNA extraction and 16S rRNA qPCR

Once thawed, DNA extraction was performed on the biofilm suspensions using the DNeasy Ultra-Clean Microbial kit (Qiagen, LOT: 169011985) according to the manufacturer’s instructions. To extract DNA directly from the particles, cryogenic aliquots of inoculated particles were thawed, and DNA extraction was performed using the DNeasy PowerBiofilm kit (Qiagen, LOT: 169048148) according to the manufacturer’s instructions. These DNA samples were then used as template DNA for 16S rRNA quantitative polymerase chain reaction (qPCR).

Standard curves were generated with custom synthetic gBlocks (Table 2) provided by IDTDNA, prepared according to the manufacturer’s instructions and stored in single-use aliquots at-20°C. gBlocks used to generate standard curves were diluted 10-fold from 10^6^ to 10^2^. Efficiency of qPCR reactions ranged from 91% to 95%, with R^2^ values ranging from 0.997–0.999. qPCR was performed using the PrimerDesign Precision Plus SYBR Green Master Mix (Z-PPLUS-R-SY-10ML), on the Applied Biosystems QuantStudio 7 Flex (serial number 278871498). Reactions comprised of 10 μL Master Mix, 5 μL template, 2 μL primer (1 μL of each forward and reverse primers, Table 2), 0.2 μL bovine serum albumin (20 mg/mL, Fisher Scientific, LOT, 170419–0461), and nuclease free water up to a final volume of 20 μL. Cycling parameters used were a 2-min initial Hot Start activation at 95°C, followed by 40 cycles of data collection with 10 s at 95°C and 60 s at 60°C. Primers are listed in Table 2 and were provided by IDTDNA.

#### 3.2.5. Statistics

All statistics were performed in RStudio. All data (CFU/mL, OD readings and 16 s rRNA gene copy number, inclusive) were tested for normality using a Shapiro Wilks test. Where data was normally distributed, a t-test was used to compare biofilm extraction methods. Where data was non-normally distributed, a non-parametric Kruskal Wallis test was used. Where *p* < 0.05, a Dunn’s test was further used to identify significant differences. *p*-values were adjusted for multiple testing.

## 4. Results

**Figure 3:**
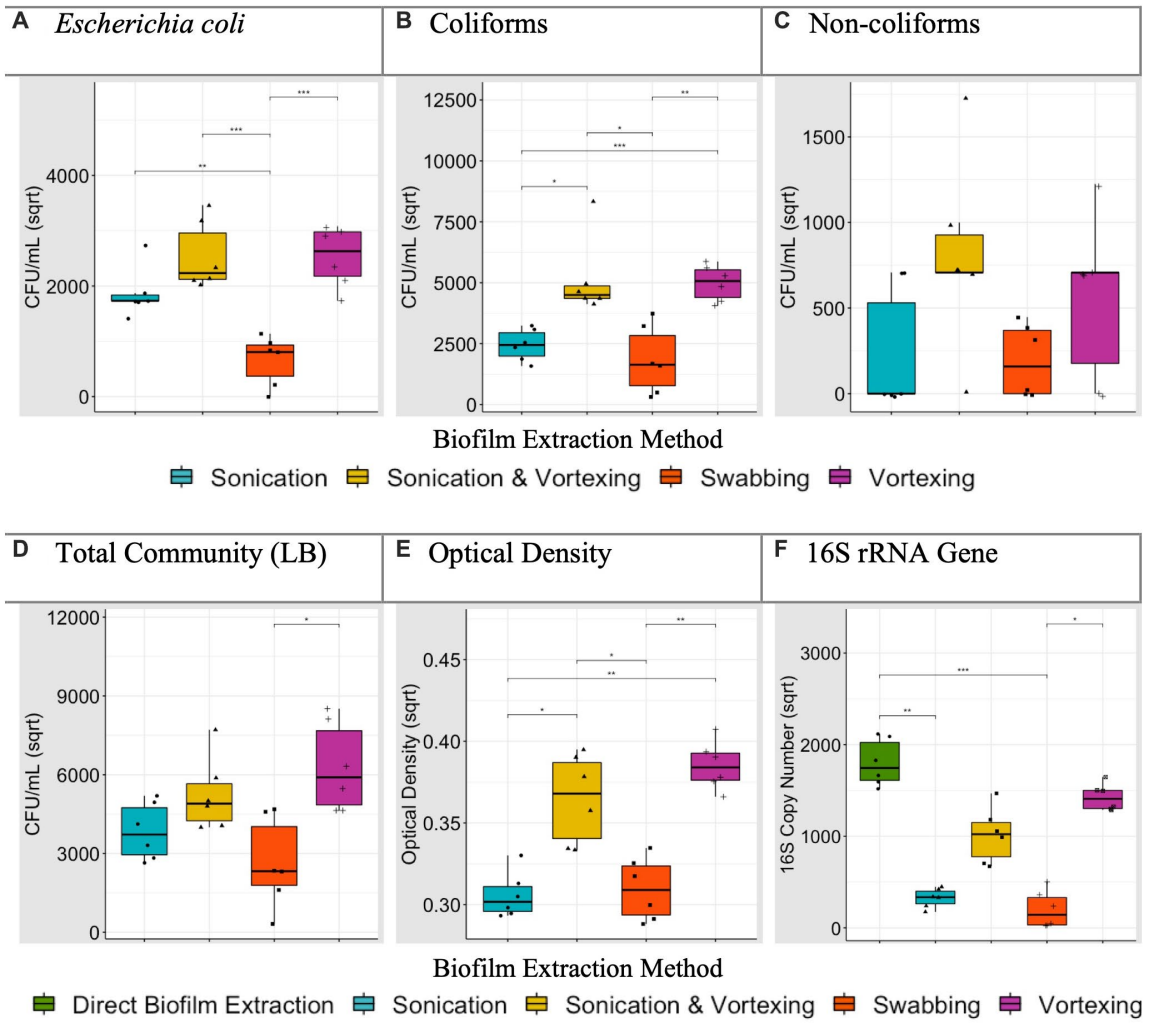
Average (biological replicate = 6) polystyrene biofilm extraction quantification for each extraction technique. ^*^p < 0.05, *t*-test or Dunn’s test according to normality (adjusted for multiple comparisons). Plots **(A–D)** present square root transformed CFU/mL data. Plot **(E)** presents square root transformed OD (600 nm) data. Plot **(F)** presents square root transformed 16S rRNA gene copy number data, including the direct biofilm DNA extraction treatment.

For the different particle types, CFU/mL were compared for each biofilm extraction method and verified against OD readings and 16S rRNA qPCR. Specifically, we investigated which extraction method significantly yielded the greatest *E. coli,* coliform, non-coliform and LB (‘total bacteria’) CFU/mL and highest OD readings. For 16S rRNA data, we determined which extraction method achieved an average 16S rRNA gene copy number that was not significantly different to (or greater than) the direct biofilm DNA extraction control. Where an extraction method, or multiple extraction methods, were found to be the most efficient, this was noted in Table 3. For example, data generated for polystyrene (Figure 3), revealed that sonication followed by vortexing, and vortexing alone yielded coliform counts significantly higher than sonication alone, or swabbing (*p* < 0.05). In this case, sonication followed by vortexing and vortexing alone were allocated as the most efficient extraction methods for coliforms (Table 3).

**Table 3:** Summary table of results.

Extraction method	Quantification type	Particle				
		Bio-bead	Nurdle	Polystyrene	Wood	Glass
*Swabbing*	*E. coli*					
	Coliforms					
	Non-coliforms					
	Total (LB)					
	OD					*x*
	16S rRNA					
*Vortexing with glass beads*	*E. coli*		*x*	*x*		*x*
	Coliforms		*x*	*x*		*x*
	Non-coliforms	*x*				
	Total (LB)		*x*	*x*		
	OD			*x*		
	16S rRNA	*x*	*x*	*x*	*x*	*x*
*Sonicating with glass beads*	*E. coli*	*x*	*x*	*x*		*x*
	Coliforms	*x*	*x*			*x*
	Non-coliforms	*x*	*x*			
	Total (LB)	*x*	*x*			*x*
	OD	*x*				
	16S rRNA		*x*			
*Sonicating, then vortexing with glass beads*	*E. coli*	*x*	*x*	*x*	*x*	*x*
	Coliforms	*x*	*x*	*x*	*x*	*x*
	Non-coliforms	*x*	*x*		*x*	
	Total (LB)	*x*	*x*		*x*	*x*
	OD			*x*	*x*	
	16S rRNA	*x*	*x*	*x*	*x*	*x*

Green (*x*) = most efficient extraction method, i.e., achieved the greatest removal of bacteria from the particle-associated biofilms. *E. coli*, *Escherichia coli*; LB, Luria-Bertani; OD, optical density; rRNA, ribosomal ribonucleic acid.

After analysing biofilm extraction data for all particles (all Figures for remaining particles can be found in Supplementary material, summarised in Table 3), swabbing was the most efficient extraction method 1 time (3.3%), vortexing with glass beads was the most efficient 15 times (50%), sonicating with glass beads was the most efficient 14 times (47%) and sonicating followed by vortexing with glass beads was the most efficient 24 times (80%; Table 3). With this, and taking the processing time (Table 4) into account, we concluded that sonication followed by vortexing was the most efficient method for extracting the biofilms from our study particles.

**Table 4:** Time taken to complete biofilm extraction processes per extraction method.

	Sonication	Sonication followed by vortexing	Vortexing	Swabbing
*Time (h)*	1	1.5	1	4

## 5. Discussion

This study aimed to identify a simple and repeatable method which efficiently extracted particle-associated biofilm communities into a culturable, bacterial suspension. To do this, we used four different extraction methods, which had been previously adopted (Table 1) to extract complex bacterial biofilms from inoculated microplastics and control particles. By comparing CFU/mL, OD readings and 16S rRNA qPCR and factoring in time constraints, we concluded that sonication with glass beads followed by vortexing was the most efficient way to extract the biofilms from our test particles (bio-beads, nurdles, polystyrene, wood and glass).

Supporting our finding, Kobayashi et al. (2009) performed a similar study which aimed to improve the detection of biofilm-forming *Staphylococcus aureus* strains on polymethylmethacrylate coupons. The combination of sonication and vortexing enhanced the yield of culturable bacteria. In addition, whilst the present study is designed with environmental microbiology in mind where bacteria grow in complex communities, Kobayashi et al. (2009) proposed that their finding may be useful in the clinical setting for dislodging biofilm forming bacteria from orthopaedic devices or other implants.

Though the primary focus of this study was to compare the efficiency of different extraction methods to collect viable cells from microplastic-associated biofilms, as a result, we also compared amount of the 16S rRNA gene. Our results suggest that a biofilm suspension step, prior to DNA extraction, is unnecessary. This is because the 16S rRNA copy numbers within the direct DNA extractions was consistently greater than most of the extraction methods we tested, irrespective of particle type. Additionally, if the biofilm suspensions are plated on agar and the cultured bacteria are used for downstream molecular work, it is important to recognise that the majority of naturally occurring bacteria are non-culturable, and therefore this would not give a full community representation.

As for the limitations in the present study, it should be noted that the methods described here may only be suitable for larger microplastic particles, greater than 2 mm, and of an appropriate size for sonication and vortexing. Additional modifications may be required for smaller particles, and for larger particles, methods like swabbing and scraping may be more suitable. Finally, culturing bacteria from nanoplastics remains largely uninvestigated and requires further research attention. When investigating the role of nanoplastics in supporting Plastisphere communities, unique challenges arise, largely owing to the smaller dimensions of these particles, which may be similar to or even smaller than microorganisms (Shi et al., 2023). Therefore, though nanoplastics are proposed to pose greater levels of toxicity than microplastics (Sharma et al., 2023), their ability to support microbial communities or influence interactions between microbes is unclear, and methods to investigate this remain under development.

In summary, we demonstrate that using sonication in combination with vortexing produces an efficient yield of culturable cells, comparable to the quantity that can be obtained by extracting the DNA directly from the biofilm using a specifically designed, biofilm extraction kit. We therefore propose that sonication in combination with vortexing should be adopted when isolating culturable bacteria from the Plastisphere. By standardising our methods across laboratories and study designs, it will increase our understanding of the role of microplastics in supporting distinct, pathogenic or AMR communities, and the subsequent ecological threats they pose.

## Data availability statement

The original contributions presented in the study are included in the article/Supplementary material, further inquiries can be directed to the corresponding author.

## Author contributions

ES: Conceptualization, Data curation, Formal analysis, Funding acquisition, Investigation, Methodology, Validation, Visualization, Writing – original draft, Writing – review & editing. AB: Conceptualization, Funding acquisition, Methodology, Supervision, Validation, Writing – review & editing. MC: Conceptualization, Funding acquisition, Methodology, Supervision, Validation, Writing – review & editing. PL: Conceptualization, Funding acquisition, Methodology, Supervision, Validation, Writing – review & editing. AM: Conceptualization, Funding acquisition, Methodology, Project administration, Supervision, Validation, Writing – review & editing.

## Funding

The author(s) declare financial support was received for the research, authorship, and/or publication of this article. We acknowledge support and give thanks to the generous philanthropic donations made to support this work from Melissa Murdoch, the Barnsbury Trust and Beach Guardian. Thank you also to the University of Exeter and Plymouth Marine Laboratory for providing funding for this research. AB acknowledges support from NERC and BBSRC. AM acknowledges support from NERC (NE/W006251/1). PL and MC acknowledge support from NERC (NE/V007351/1).

## Conflict of interest

The authors declare that the research was conducted in the absence of any commercial or financial relationships that could be construed as a potential conflict of interest.

## Publisher’s note

All claims expressed in this article are solely those of the authors and do not necessarily represent those of their affiliated organizations, or those of the publisher, the editors and the reviewers. Any product that may be evaluated in this article, or claim that may be made by its manufacturer, is not guaranteed or endorsed by the publisher.
