# Supplementary material for: Culturing the Plastisphere: comparing methods to isolate culturable bacteria colonising microplastics
Source: Front Microbiol. 2023 Oct 3;14:1259287. doi: 10.3389/fmicb.2023.1259287 (PMC10579789; doi:10.3389/fmicb.2023.1259287)
Supplement: Supplementary file 1 [file Data_Sheet_1.pdf]

Supplementary Material

Culturing the Plastisphere: comparing methods to isolate culturable bacteria colonising microplastics

Emily M Stevenson, Angus Buckling, Matthew Cole, Penelope K Lindeque, Aimee K Murray*

*** Correspondence:** A. K. Murray. a.k.murray@exeter.ac.uk

1.1. Supplementary Data – Biofilm Extraction Quantification

Extraction method	Particle	16s rRNA gene copy no.	*E. coli* (CFU/mL)	Coliforms (CFU/mL)	Non-coliforms (CFU/mL)	Total (LB) (CFU/mL)	Optical Density (600nm)
SO	bio-bead	12,294.490	590000	3650000	20000	4550000	0.144
SO	bio-bead	5,457.296	400000	1075000	20000	1545000	0.078
SO	bio-bead	6,499.410	445000	1490000	100000	2355000	0.085
SO	bio-bead	4,952.158	315000	2110000	50000	2620000	0.106
SO	bio-bead	5,317.146	635000	4640000	75000	6150000	0.232
SO	bio-bead	11,180.947	210000	1460000	15000	2460000	0.413
SO	glass	706.362	35000	210000	0	400000	0.077
SO	glass	927.901	20000	180000	5000	280000	0.078
SO	glass	2,251.371	30000	185000	5000	380000	0.075
SO	glass	2,994.221	10000	150000	0	235000	0.077
SO	glass	1,002.252	30000	285000	0	660000	0.077
SO	glass	412.042	15000	195000	0	275000	0.079
SO	nurdle	10,542.692	50000	1070000	25000	725000	0.081
SO	nurdle	3,528.978	65000	830000	25000	755000	0.076
SO	nurdle	1,372.471	95000	725000	15000	1235000	0.077
SO	nurdle	5,754.462	135000	1705000	0	1260000	0.086
SO	nurdle	2,935.920	310000	3015000	60000	2860000	0.079
SO	nurdle	3,612.969	105000	3530000	30000	4180000	0.084
SO	polystyrene	114,441.109	3500000	2500000	0	8000000	0.093
SO	polystyrene	109,413.625	3000000	6500000	0	11000000	0.087
SO	polystyrene	176,855.250	3000000	9500000	500000	27000000	0.109
SO	polystyrene	57,426.641	2000000	5500000	0	17000000	0.086
SO	polystyrene	200,282.969	7500000	10500000	500000	24500000	0.098
SO	polystyrene	30,488.754	3000000	3500000	0	7000000	0.089
SO	wood	858,673.250	14900000	10050000	400000	51400000	0.199
SO	wood	720,874.500	9350000	8600000	500000	23950000	0.148

SO	wood	832,165.125	23400000	17500000	950000	54400000	0.172
SO	wood	775,408.250	18900000	12100000	200000	34600000	0.178
SO	wood	839,700.125	34050000	19800000	1700000	64600000	0.191
SO	wood	840,792.125	14000000	12850000	300000	54700000	0.184
SO_VX	bio-bead	9,178.086	200000	1850000	50000	2700000	0.083
SO_VX	bio-bead	38,055.875	750000	3250000	200000	4750000	0.084
SO_VX	bio-bead	77,587.734	250000	2850000	0	2200000	0.091
SO_VX	bio-bead	104,689.453	600000	2500000	100000	3350000	0.165
SO_VX	bio-bead	15,931.453	450000	2450000	50000	2250000	0.155
SO_VX	bio-bead	33,428.727	350000	1750000	0	3650000	0.11
SO_VX	glass	882.251	10000	85000	0	75000	0.077
SO_VX	glass	6,271.783	90000	555000	0	615000	0.077
SO_VX	glass	1,278.626	25000	100000	5000	120000	0.076
SO_VX	glass	2,744.943	30000	140000	10000	80000	0.076
SO_VX	glass	1,764.421	25000	105000	0	155000	0.077
SO_VX	glass	1,068.692	15000	40000	0	265000	0.078
SO_VX	nurdle	38,754.000	350000	3600000	35000	3570000	0.14
SO_VX	nurdle	22,015.008	430000	4040000	50000	4810000	0.085
SO_VX	nurdle	2,994.192	75000	780000	5000	950000	0.078
SO_VX	nurdle	8,702.727	90000	1050000	20000	1130000	0.08
SO_VX	nurdle	2,943.829	90000	1165000	15000	585000	0.078
SO_VX	nurdle	143.117	110000	1780000	25000	1505000	0.086
SO_VX	polystyrene	451,730.188	4000000	19000000	500000	23000000	0.111
SO_VX	polystyrene	2,154,113.250	12000000	69500000	3000000	59500000	0.156
SO_VX	polystyrene	1,394,487.000	10000000	24500000	500000	25000000	0.152
SO_VX	polystyrene	1,113,545.750	4500000	17000000	0	34500000	0.143
SO_VX	polystyrene	497,821.844	4500000	21500000	1000000	16000000	0.112
SO_VX	polystyrene	979,114.063	5500000	19000000	500000	16500000	0.128
SO_VX	wood	4,883,375.000	107500000	82000000	3500000	251000000	0.296
SO_VX	wood	3,990,497.000	55500000	60500000	3000000	128000000	0.276
SO_VX	wood	2,866,259.000	43000000	55000000	1000000	97500000	0.287
SO_VX	wood	3,831,689.000	63500000	105500000	2000000	137500000	0.295
SO_VX	wood	4,048,934.500	60500000	39000000	4000000	92500000	0.334
SO_VX	wood	3,989,411.750	37000000	36000000	1500000	90500000	0.259
SW	bio-bead	329.582	16000	48500	1500	86000	0.093
SW	bio-bead	205.983	4500	35000	2000	68000	0.094
SW	bio-bead	305.271	1000	23000	0	57000	0.084
SW	bio-bead	334.998	20000	75500	3000	153000	0.085
SW	bio-bead	162.345	14000	30500	1500	66000	0.084

SW	bio-bead	1,210.809	30000	165500	6000	206000	0.089
SW	glass	3.650	0	0	0	0	0.085
SW	glass	6.874	1050	900	50	2550	0.084
SW	glass	28.385	300	250	50	0	0.086
SW	glass	54.369	1350	3700	50	5850	0.081
SW	glass	13.745	350	450	0	650	0.084
SW	glass	24.430	1750	7900	250	10550	0.086
SW	nurdle	8.432	0	0	0	0	0.083
SW	nurdle	56.336	500	0	0	1000	0.092
SW	nurdle	95.482	1000	2000	0	7000	0.082
SW	nurdle	10.874	0	0	0	500	0.083
SW	nurdle	42.964	0	3000	0	3500	0.086
SW	nurdle	26.881	5000	21500	0	53500	0.082
SW	polystyrene	605.868	0	100000	0	100000	0.083
SW	polystyrene	711.905	650000	2800000	0	5450000	0.09
SW	polystyrene	56,272.250	650000	2550000	100000	5350000	0.112
SW	polystyrene	131,428.344	1300000	10400000	200000	21050000	0.101
SW	polystyrene	2,283.512	50000	250000	0	2600000	0.085
SW	polystyrene	253,926.344	950000	13950000	150000	21950000	0.106
SW	wood	100,232.320	250000	350000	0	4400000	0.288
SW	wood	24,573.465	150000	850000	0	1350000	0.13
SW	wood	28,036.297	250000	300000	0	2800000	0.1
SW	wood	53,642.969	100000	200000	0	2850000	0.113
SW	wood	1,504.344	0	0	0	0	0.096
SW	wood	153,650.406	250000	1650000	0	3900000	0.131
VX	bio-bead	2,628.823	130000	1000000	15000	1930000	0.081
VX	bio-bead	28,660.105	180000	985000	35000	1265000	0.083
VX	bio-bead	34,430.406	155000	550000	15000	1145000	0.083
VX	bio-bead	36,649.297	270000	1200000	20000	2015000	0.083
VX	bio-bead	41,169.406	285000	1155000	25000	2395000	0.082
VX	bio-bead	15,239.771	255000	755000	20000	735000	0.078
VX	glass	1,720.474	10000	49000	500	81500	0.076
VX	glass	2,915.567	15500	65000	1000	93500	0.076
VX	glass	4,101.794	26500	160000	3000	237000	0.076
VX	glass	5,072.094	42000	117000	2500	214500	0.077
VX	glass	3,062.327	27500	198000	6500	216500	0.075
VX	glass	4,735.444	40500	105000	6000	185500	0.077
VX	nurdle	24,528.738	60000	625000	5000	1110000	0.078
VX	nurdle	44,646.285	40000	410000	0	1130000	0.078
VX	nurdle	21,564.754	90000	925000	10000	1170000	0.078

VX	nurdle	45,193.195	20000	720000	0	1130000	0.081
VX	nurdle	38,111.922	140000	1495000	10000	1715000	0.08
VX	nurdle	9,009.746	85000	715000	15000	755000	0.08
VX	polystyrene	1,682,875.625	5500000	16500000	500000	21500000	0.141
VX	polystyrene	2,708,994.000	9000000	23500000	1500000	30000000	0.152
VX	polystyrene	1,744,552.750	3000000	18000000	0	21500000	0.134
VX	polystyrene	2,255,564.000	9500000	31500000	500000	66000000	0.155
VX	polystyrene	1,658,603.000	4500000	28000000	0	40000000	0.143
VX	polystyrene	2,237,409.750	8500000	34500000	500000	72500000	0.166
VX	wood	5,904,698.500	19000000	32500000	0	40500000	0.193
VX	wood	4,619,487.500	27000000	21000000	500000	78500000	0.189
VX	wood	6,180,857.000	34500000	29000000	1000000	63000000	0.24
VX	wood	4,172,522.000	26500000	23000000	500000	32500000	0.17
VX	wood	6,941,594.500	47500000	32000000	1000000	66000000	0.223
VX	wood	6,380,523.500	23000000	24000000	0	71500000	0.209
B	bio-bead	127,368.691					
B	bio-bead	237,872.539					
B	bio-bead	130,309.668					
B	bio-bead	118,107.305					
B	bio-bead	201,059.883					
B	bio-bead	118,429.688					
B	glass	5,287.200					
B	glass	5,679.250					
B	glass	6,703.537					
B	glass	5,445.432					
B	glass	5,105.645					
B	glass	5,571.639					
B	nurdle	62,554.531					
B	nurdle	59,890.752					
B	nurdle	38,116.602					
B	nurdle	33,977.324					
B	nurdle	63,322.031					
B	nurdle	31,917.612					
B	polystyrene	4,475,641.250					
B	polystyrene	4,363,658.750					
B	polystyrene	2,765,854.375					
B	polystyrene	2,537,332.656					
B	polystyrene	3,338,083.750					
B	polystyrene	2,304,323.906					

B	wood	1,800,284.844					
B	wood	944,705.781					
B	wood	1,101,047.813					
B	wood	1,380,516.563					
B	wood	2,494,430.938					
B	wood	2,310,567.969					

* B: direct biofilm DNA extraction, SO: sonication, SO_VX: sonication and vortexing, VX: vortexing.

1.2. Supplementary Data – Comparison of Blanks Between Plate Readers

**Plate Reader Model**	**Optical Density (600nm)**					
	Replicate 1	*Replicate 2*	*Replicate 3*	*Replicate 4*	*Replicate 5*	*Replicate 6*
Varioskan Flash	0.036	0.036	0.036	0.037	0.036	0.036
BioTek Synergy	0.036	0.037	0.038	0.038	0.036	0.036

2 Supplementary Figures

2.1. Bio-bead

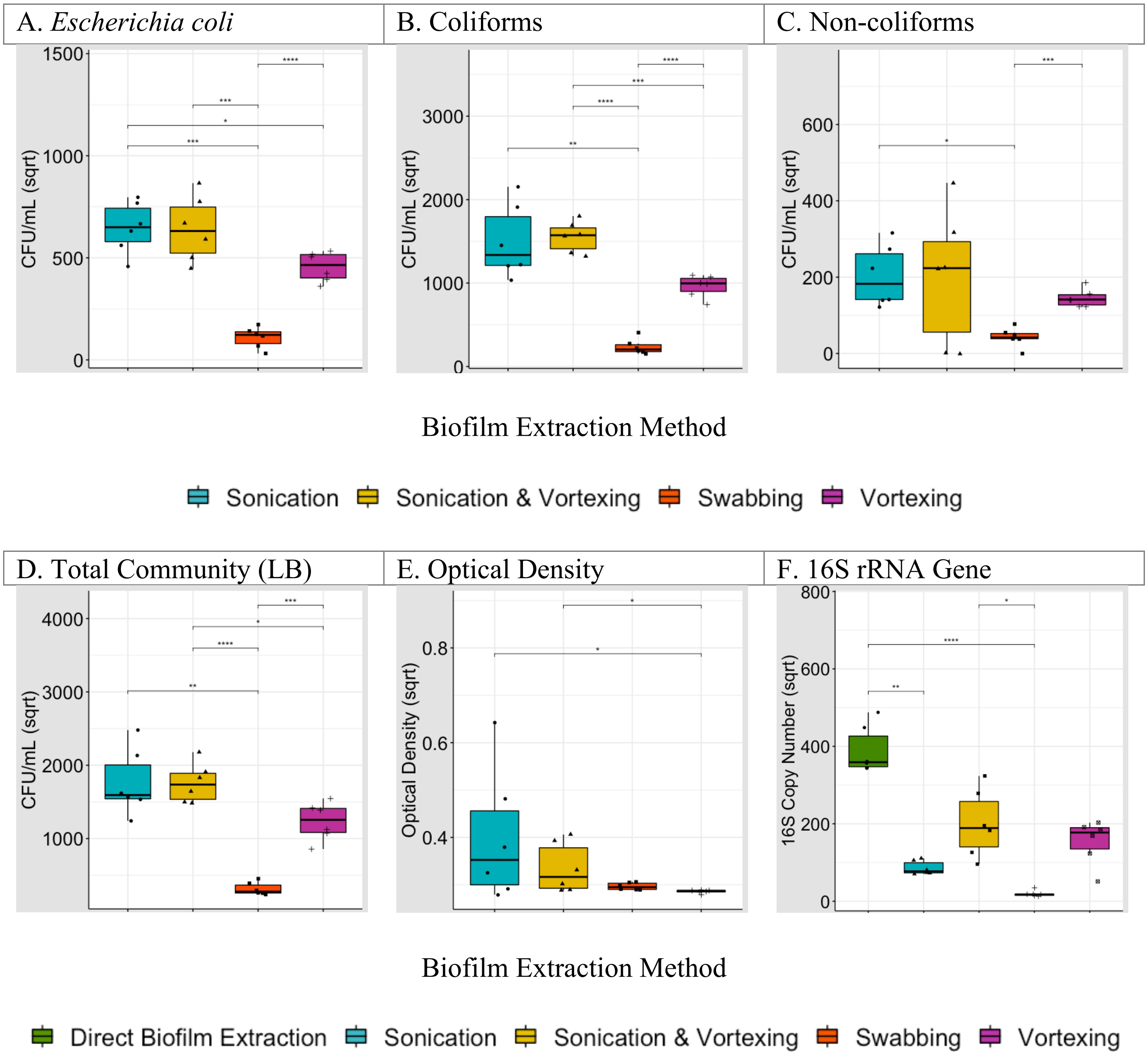

Supplementary Figure 1. Average (biological replicate = 6) bio-bead biofilm extraction quantification for each extraction technique. *: $p < 0.05$, t-test or Dunn's test according to normality (adjusted for multiple comparisons). Plots A-D present square root transformed CFU/mL data. Plot E presents square root transformed OD (600nm) data. Plot F presents square root transformed 16S rRNA gene copy number data, including the direct biofilm DNA extraction treatment.

2.2. Nurdles

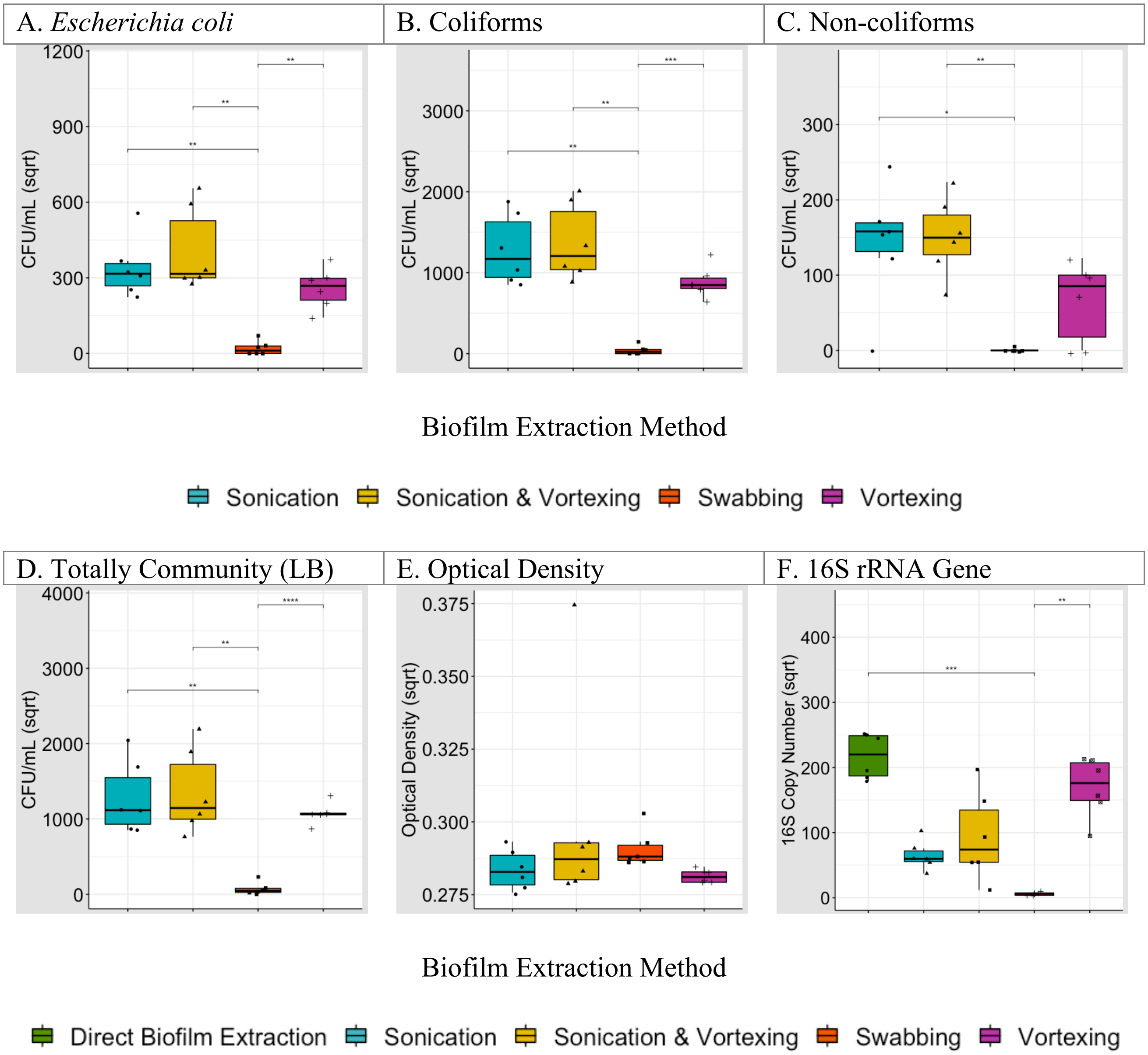

Supplementary Figure 2. Average (biological replicate = 6) nurdle biofilm extraction quantification for each extraction technique. *: $p < 0.05$, t-test or Dunn's test according to normality (adjusted for multiple comparisons). Plots A-D present square root transformed CFU/mL data. Plot E presents square root transformed OD (600nm) data. Plot F presents square root transformed 16S rRNA gene copy number data, including the direct biofilm DNA extraction treatment.

2.3. Glass

A. *Escherichia coli*	B. Coliforms	C. Non-coliforms

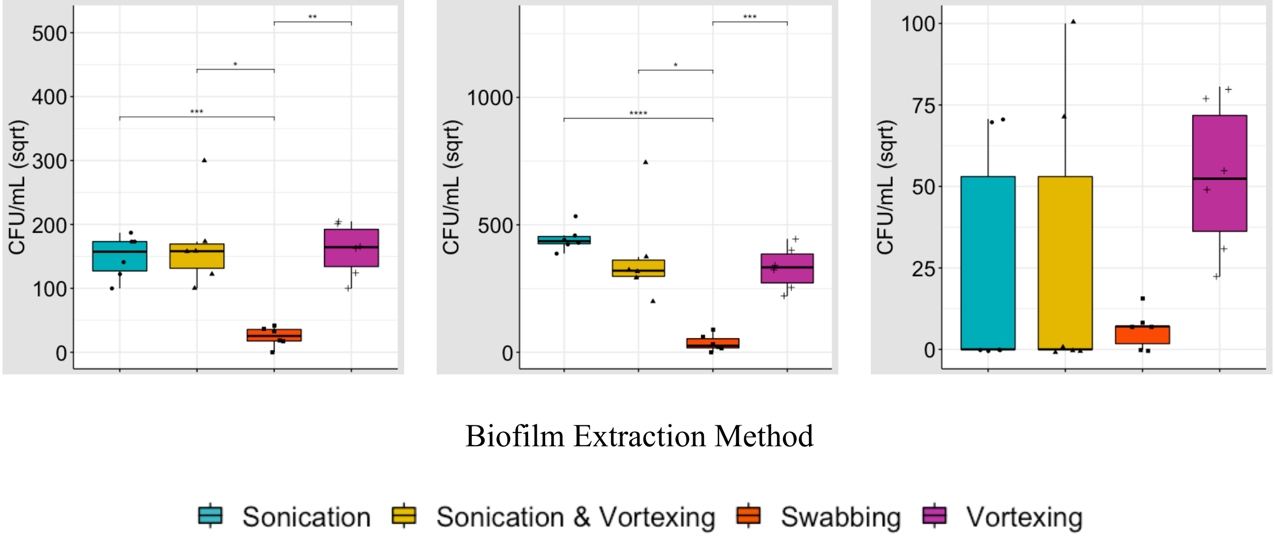

D. Totally Community (LB)	E. Optical Density	F. 16S rRNA Gene

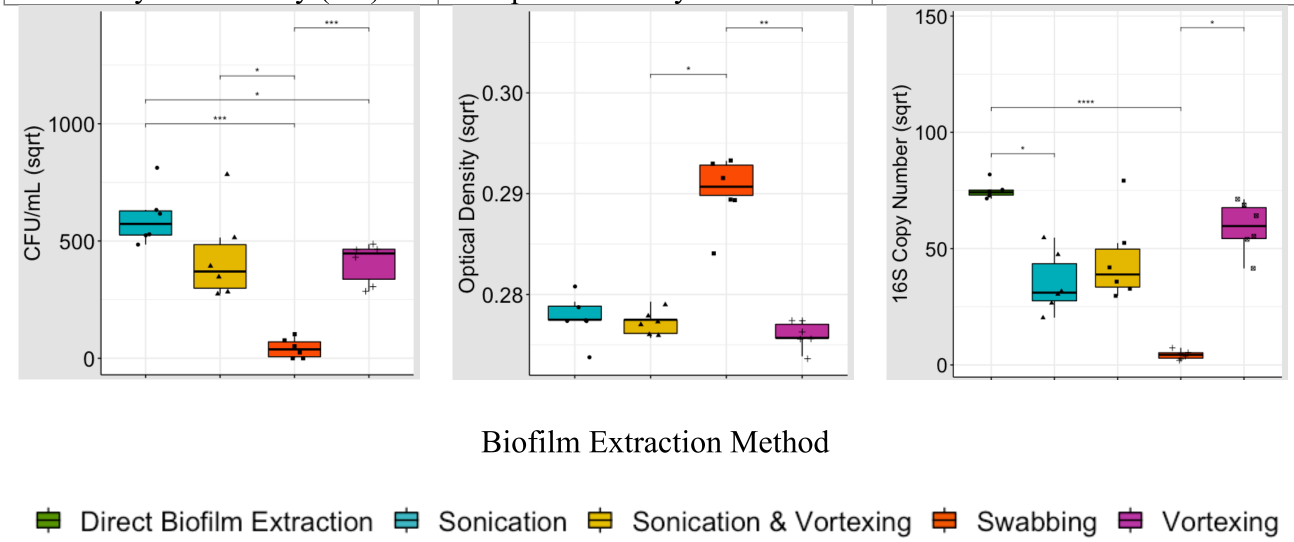

Supplementary Figure 3. Average (biological replicate = 6) glass biofilm extraction quantification for each extraction technique. *: $p < 0.05$, t-test or Dunn's test according to normality (adjusted for multiple comparisons). Plots A-D present square root transformed CFU/mL data. Plot E presents square root transformed OD (600nm) data. Plot F presents square root transformed 16S rRNA gene copy number data, including the direct biofilm DNA extraction treatment.

2.4. Wood

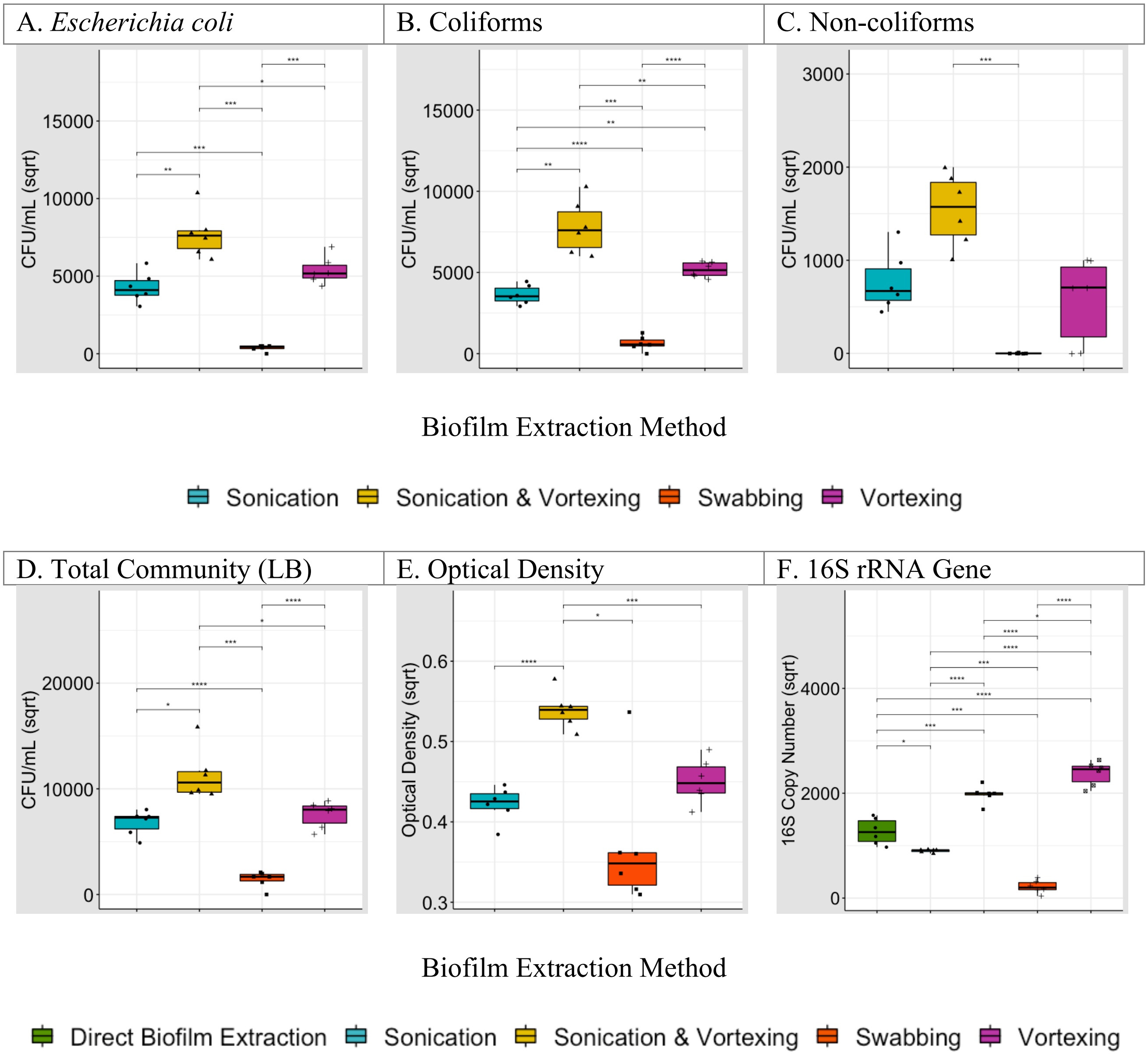

Supplementary Figure 4. Average (biological replicate = 6) wood biofilm extraction quantification for each extraction technique. *: $p < 0.05$, t-test or Dunn's test according to normality (adjusted for multiple comparisons). Plots A-D present square root transformed CFU/mL data. Plot E presents square root transformed OD (600nm) data. Plot F presents square root transformed 16S rRNA gene copy number data, including the direct biofilm DNA extraction treatment.
